# Supplementary material for: Combination Therapy with an SGLT2 Inhibitor as Initial Treatment for Type 2 Diabetes: A Systematic Review and Meta-Analysis
Source: J Clin Med. 2019 Jan 4;8(1):45. doi: 10.3390/jcm8010045 (PMC6352265; doi:10.3390/jcm8010045)
Supplement: Supplementary file 1 [file jcm-08-00045-s001.pdf]

Supplementary material

Table S1. Search strategy performed on the following databases: PubMed, Embase, the Cochrane Central Register of Controlled Trials (CENTRAL).

1. Randomi*ed study OR random allocation OR Randomi*ed controlled trial OR Random* Control* trial OR RCT Epidemiological study
2. sodium glucose cotransporter 2 OR sodium glucose cotransporter 2 inhibitor* OR sglt2 inhibitor* OR empagliflozin OR dapagliflozin OR canagliflozin OR ipragliflozin OR tofogliflozin OR ertugliflozin OR sotagliflozin OR sergliflozin OR remogliflozin
3. 1 AND 2

Table S2. Safety outcomes of empagliflozin and linagliptin combination therapy compared with empagliflozin or linagliptin monotherapy in treatment naïve type 2 diabetes patients

Safety outcome	Comparator 1	Comparator 2	I²	RR [95% CI]
	Number of events / total subjects	Number of events / total subjects		
**i. Empagliflozin + linagliptin vs empagliflozin monotherapy**				
	Empagliflozin + linagliptin	*Empagliflozin monotherapy*		
≥ 1 AE(s)	202/272	203/270	77%	0.99 [0.81, 1.21]
≥ 1 drug-related AE(s)	37/272	38/270	0%	0.97 [0.64, 1.47]
≥ 1 serious AE(s)	13/272	19/270	0%	0.68 [0.34, 1.35]
Hypoglycaemia*	0/272	5/270	0%	0.18 [0.02, 1.56]
UTI	32/272	25/270	29%	1.28 [0.70, 2.35]
Events suggestive of genital infection	12/272	13/270	9%	0.92 [0.40, 2.09]
**i. Empagliflozin + linagliptin vs linagliptin monotherapy**				
	Empagliflozin + linagliptin	*Linagliptin monotherapy*		
≥ 1 AE(s)	202/272	97/135	0%	1.03 [0.91, 1.17]
≥ 1 drug-related AE(s)	37/272	17/135	0%	1.08 [0.63, 1.84]
≥ 1 serious AE(s)	13/272	2/135	0%	3.22 [0.74, 14.07]
Hypoglycaemia*	0/272	1/135	NA	0.17 [0.01, 4.07]
UTI	32/272	12/135	0%	1.32 [0.70, 2.49]
Events suggestive of genital infection	12/272	4/135	0%	1.45 [0.47, 4.47]

RR, relative risk; AE, adverse event; UTI, urinary tract infection. * Hypoglycaemia defined as plasma glucose ≤ 3.9 mmol/L and/or assistance required.

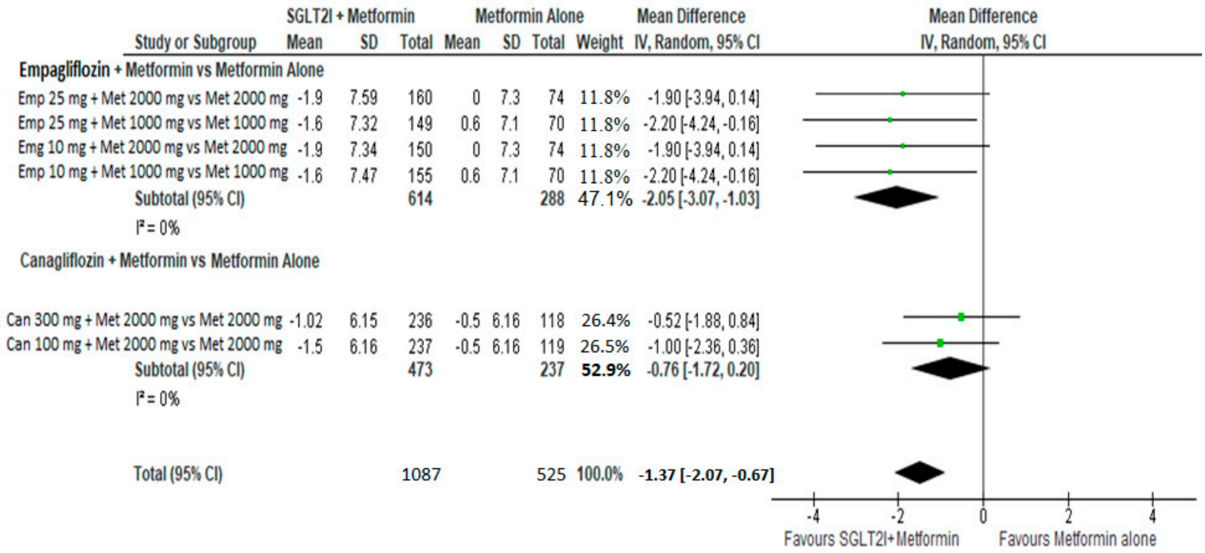

Figure S1. The mean change from baseline (pre-treatment) in diastolic BP (mmHg) between SGLT2 inhibitor and metformin combination therapy versus metformin monotherapy in treatment naïve type 2 diabetes patients. SGLT2I, SGLT2 inhibitor; Emp, empagliflozin; Met, metformin; Dap, dapagliflozin; Can, canagliflozin.

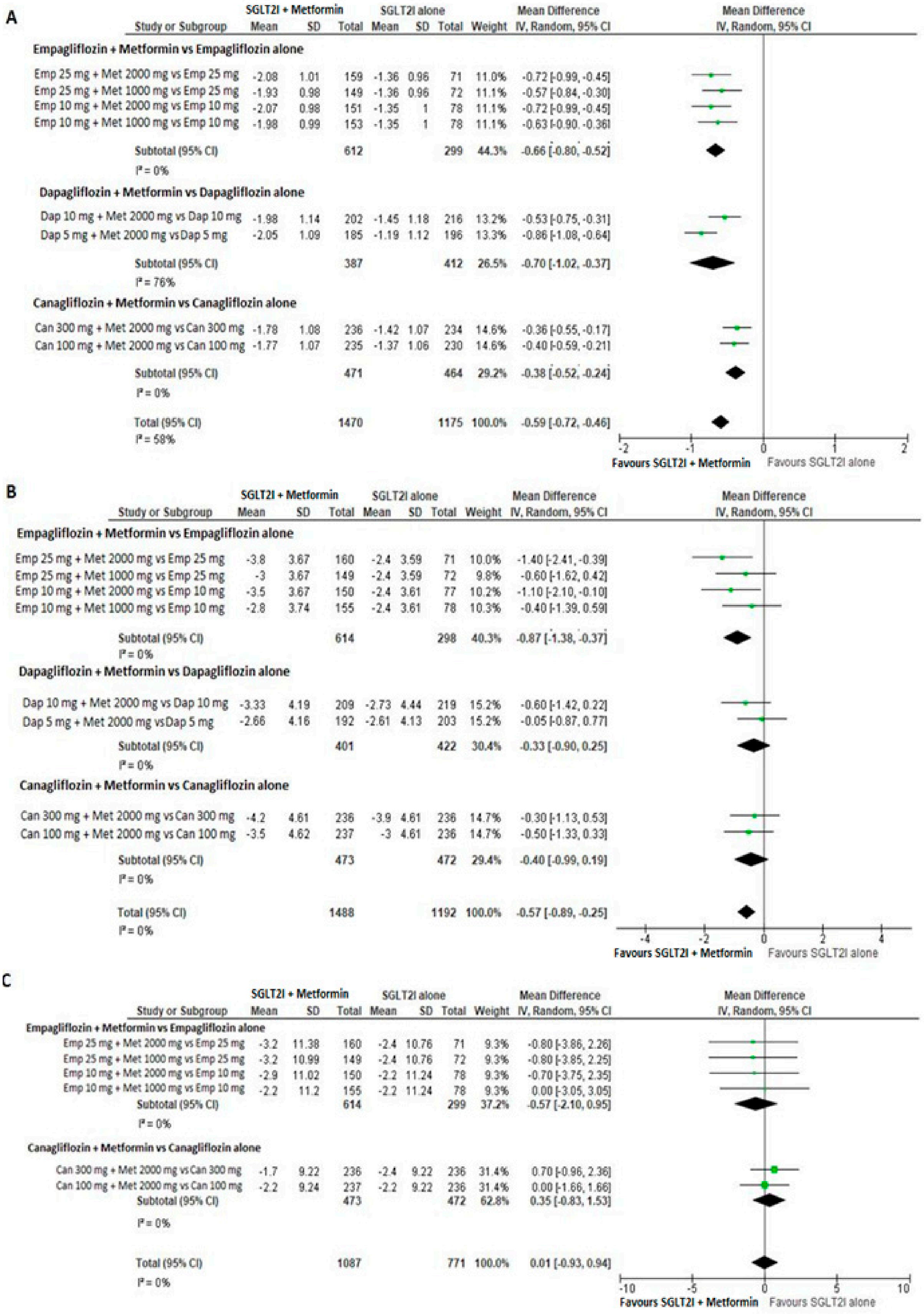
A
SGLT2I + Metformin
SGLT2I alone
Mean Difference
Mean Difference
Study or Subgroup Mean SD Total Mean SD Total Weight IV, Random, 95% CI
IV, Random, 95% CI
Empagliflozin + Metformin vs Empagliflozin alone
Emp 25 mg + Met 2000 mg vs Emp 25 mg -2.08 1.01 159 -1.36 0.96 71 11.0% -0.72 [-0.99, -0.45]
Emp 25 mg + Met 1000 mg vs Emp 25 mg -1.93 0.98 149 -1.36 0.96 72 11.1% -0.57 [-0.84, -0.30]
Emp 10 mg + Met 2000 mg vs Emp 10 mg -2.07 0.98 151 -1.35 1 78 11.1% -0.72 [-0.99, -0.45]
Emp 10 mg + Met 1000 mg vs Emp 10 mg -1.98 0.99 153 -1.35 1 78 11.1% -0.63 [-0.90, -0.36]
Subtotal (95% CI) 612 299 44.3% -0.66 [-0.80, -0.52]
I² = 0%
Dapagliflozin + Metformin vs Dapagliflozin alone
Dap 10 mg + Met 2000 mg vs Dap 10 mg -1.98 1.14 202 -1.45 1.18 216 13.2% -0.53 [-0.75, -0.31]
Dap 5 mg + Met 2000 mg vs Dap 5 mg -2.05 1.09 185 -1.19 1.12 196 13.3% -0.86 [-1.08, -0.64]
Subtotal (95% CI) 387 412 26.5% -0.70 [-1.02, -0.37]
I² = 76%
Canagliflozin + Metformin vs Canagliflozin alone
Can 300 mg + Met 2000 mg vs Can 300 mg -1.78 1.08 236 -1.42 1.07 234 14.6% -0.36 [-0.55, -0.17]
Can 100 mg + Met 2000 mg vs Can 100 mg -1.77 1.07 235 -1.37 1.06 230 14.6% -0.40 [-0.59, -0.21]
Subtotal (95% CI) 471 464 29.2% -0.38 [-0.52, -0.24]
I² = 0%
Total (95% CI) 1470 1175 100.0% -0.59 [-0.72, -0.46]
I² = 58%
-2 -1 0 1 2
Favours SGLT2I + Metformin Favours SGLT2I alone
B
SGLT2I + Metformin
SGLT2I alone
Mean Difference
Mean Difference
Study or Subgroup Mean SD Total Mean SD Total Weight IV, Random, 95% CI
IV, Random, 95% CI
Empagliflozin + Metformin vs Empagliflozin alone
Emp 25 mg + Met 2000 mg vs Emp 25 mg -3.8 3.67 160 -2.4 3.59 71 10.0% -1.40 [-2.41, -0.39]
Emp 25 mg + Met 1000 mg vs Emp 25 mg -3 3.67 149 -2.4 3.59 72 9.8% -0.60 [-1.62, 0.42]
Emp 10 mg + Met 2000 mg vs Emp 10 mg -3.5 3.67 150 -2.4 3.61 77 10.2% -1.10 [-2.10, -0.10]
Emp 10 mg + Met 1000 mg vs Emp 10 mg -2.8 3.74 155 -2.4 3.61 78 10.3% -0.40 [-1.39, 0.59]
Subtotal (95% CI) 614 298 40.3% -0.87 [-1.38, -0.37]
I² = 0%
Dapagliflozin + Metformin vs Dapagliflozin alone
Dap 10 mg + Met 2000 mg vs Dap 10 mg -3.33 4.19 209 -2.73 4.44 219 15.2% -0.60 [-1.42, 0.22]
Dap 5 mg + Met 2000 mg vs Dap 5 mg -2.66 4.16 192 -2.61 4.13 203 15.2% -0.05 [-0.87, 0.77]
Subtotal (95% CI) 401 422 30.4% -0.33 [-0.90, 0.25]
I² = 0%
Canagliflozin + Metformin vs Canagliflozin alone
Can 300 mg + Met 2000 mg vs Can 300 mg -4.2 4.61 236 -3.9 4.61 236 14.7% -0.30 [-1.13, 0.53]
Can 100 mg + Met 2000 mg vs Can 100 mg -3.5 4.62 237 -3 4.61 236 14.7% -0.50 [-1.33, 0.33]
Subtotal (95% CI) 473 472 29.4% -0.40 [-0.99, 0.19]
I² = 0%
Total (95% CI) 1488 1192 100.0% -0.57 [-0.89, -0.25]
I² = 0%
-4 -2 0 2 4
Favours SGLT2I + Metformin Favours SGLT2I alone
C
SGLT2I + Metformin
SGLT2I alone
Mean Difference
Mean Difference
Study or Subgroup Mean SD Total Mean SD Total Weight IV, Random, 95% CI
IV, Random, 95% CI
Empagliflozin + Metformin vs Empagliflozin alone
Emp 25 mg + Met 2000 mg vs Emp 25 mg -3.2 11.38 160 -2.4 10.76 71 9.3% -0.80 [-3.86, 2.26]
Emp 25 mg + Met 1000 mg vs Emp 25 mg -3.2 10.99 149 -2.4 10.76 72 9.3% -0.80 [-3.85, 2.25]
Emp 10 mg + Met 2000 mg vs Emp 10 mg -2.9 11.02 150 -2.2 11.24 78 9.3% -0.70 [-3.75, 2.35]
Emp 10 mg + Met 1000 mg vs Emp 10 mg -2.2 11.2 155 -2.2 11.24 78 9.3% 0.00 [-3.05, 3.05]
Subtotal (95% CI) 614 299 37.2% -0.57 [-2.10, 0.95]
I² = 0%
Canagliflozin + Metformin vs Canagliflozin alone
Can 300 mg + Met 2000 mg vs Can 300 mg -1.7 9.22 236 -2.4 9.22 236 31.4% 0.70 [-0.96, 2.36]
Can 100 mg + Met 2000 mg vs Can 100 mg -2.2 9.24 237 -2.2 9.22 236 31.4% 0.00 [-1.66, 1.66]
Subtotal (95% CI) 473 472 62.8% 0.35 [-0.83, 1.53]
I² = 0%
Total (95% CI) 1087 771 100.0% 0.01 [-0.93, 0.94]
I² = 0%
-10 -5 0 5 10
Favours SGLT2I + Metformin Favours SGLT2I alone

D

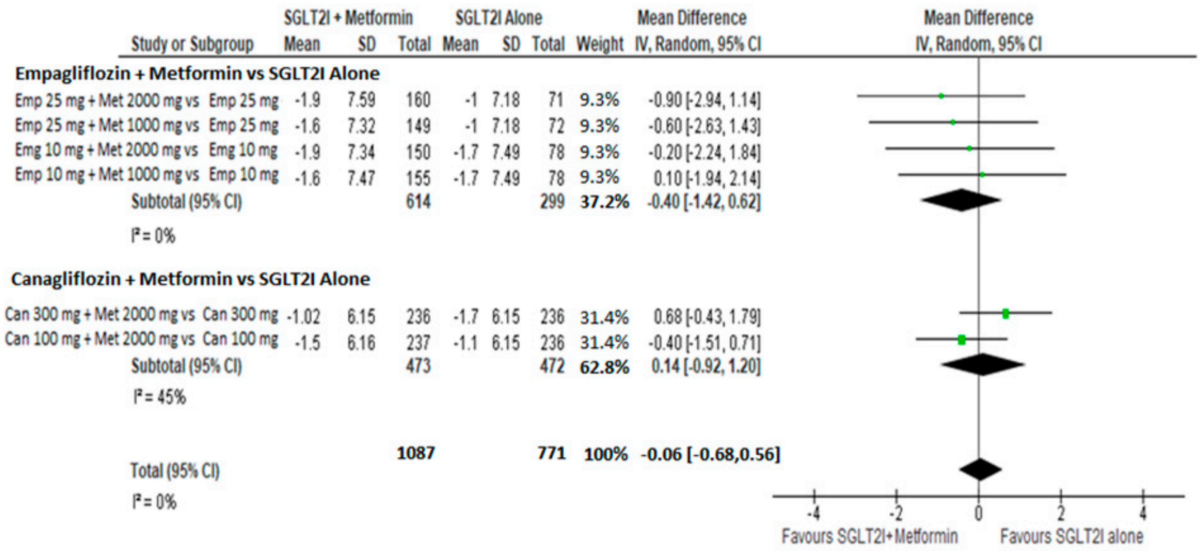

Figure S2. The mean change from baseline (pre-treatment) in: (**A**) HbA1c (%) (**B**) body weight (kg) and (**C**) systolic BP (mmHg) (**D**) diastolic BP (mmHg) between SGLT2 inhibitor and metformin combination therapy versus SGLT2 inhibitor monotherapy in treatment naïve type 2 diabetes patients. SGLT2I, SGLT2 inhibitor; Emp, empagliflozin; Met, metformin; Dap, dapagliflozin; Can, canagliflozin.

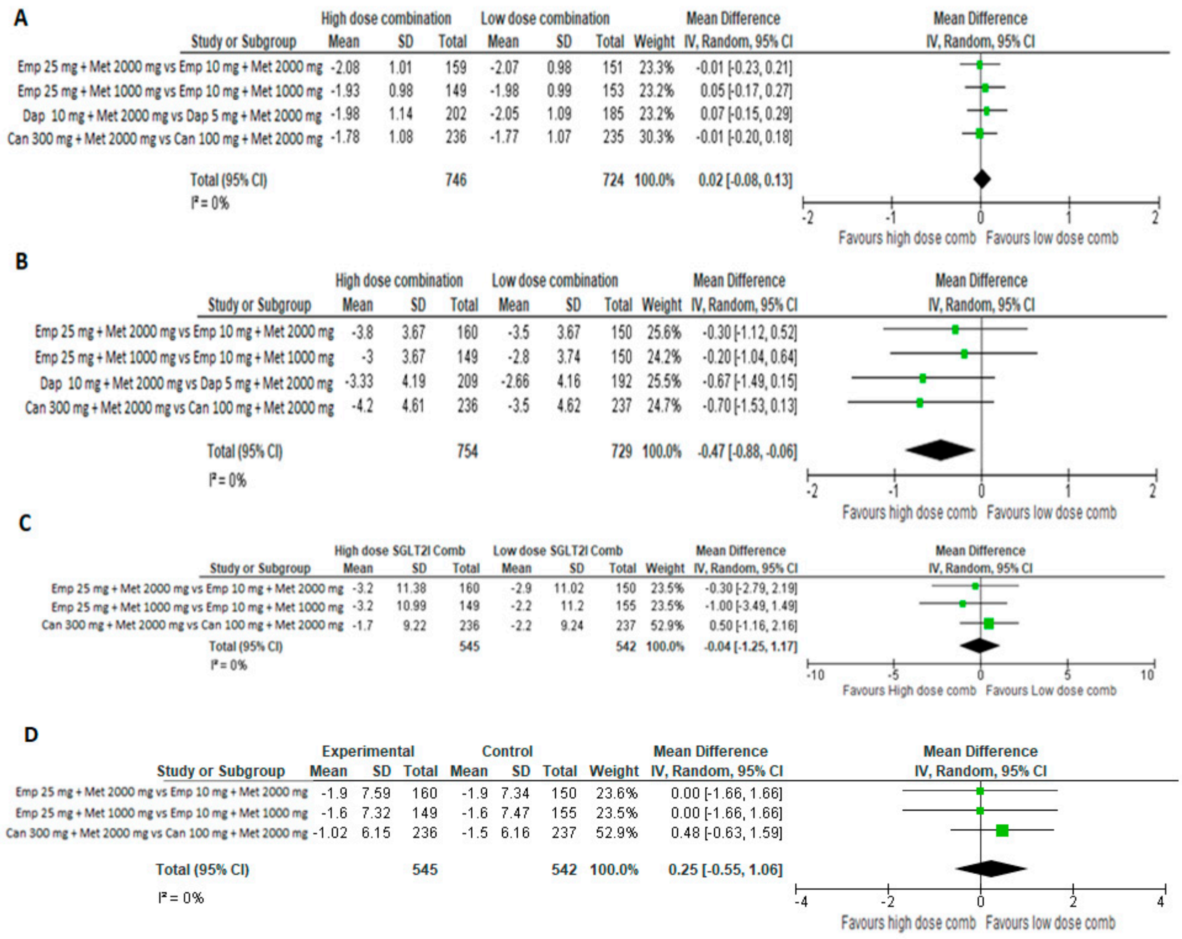

Figure S3. The mean change from baseline (pre-treatment) in: (**A**) HbA1c (%) (**B**) body weight (kg) (**C**) systolic BP (mmHg) and (**D**) diastolic BP (mmHg) between combination high dose SGLT2 inhibitor and metformin versus combination low dose SGLT2 inhibitor and metformin in treatment naïve type 2 diabetes patients. Emp, empagliflozin; Met, metformin; Dap, dapagliflozin; Can, canagliflozin.

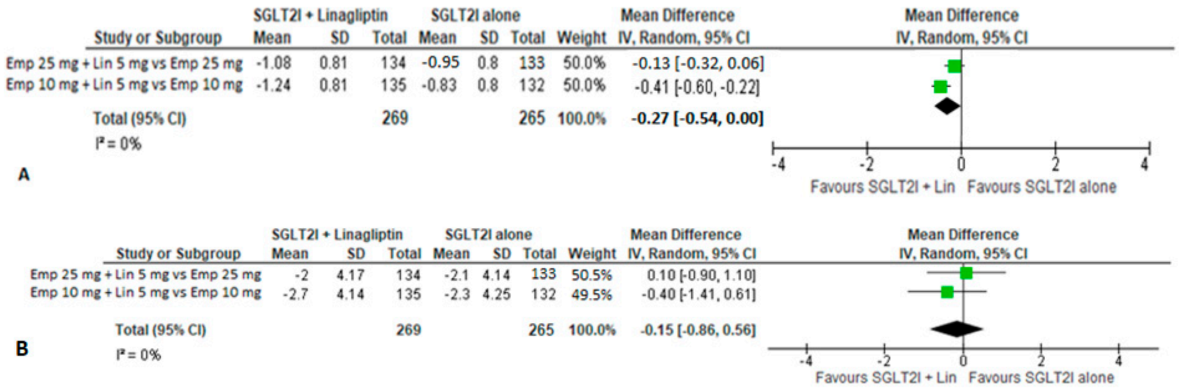

Figure S4. The mean change from baseline (pre-treatment) in: (**A**) HbA1c (%) (**B**) body weight (kg) between empagliflozin and linagliptin combination therapy versus empagliflozin monotherapy in treatment naïve type 2 diabetes patients. SGLT2I, SGLT2 inhibitor; Emp, empagliflozin; Lin, Linagliptin.

Study or Subgroup	SGLT2I + Linagliptin Mean	SD	Total	Linagliptin alone Mean	SD	Total	Weight	Mean Difference IV, Random, 95% CI
Emp 25 mg + Lin 5 mg vs Lin 5 mg	-1.08	0.81	134	-0.67	0.8	66	49.7%	-0.41 [-0.65, -0.17]
Emp 10 mg + Lin 5 mg vs Lin 5 mg	-1.24	0.81	135	-0.67	0.8	67	50.3%	-0.57 [-0.81, -0.33]
**Total (95% CI)**			**269**			**133**	**100.0%**	**-0.49 [-0.66, -0.32]**

$I^2 = 0\%$

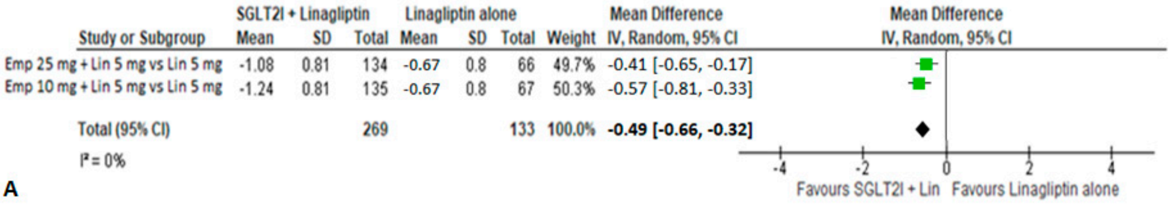

A

Study or Subgroup	SGLT2I + Linagliptin Mean	SD	Total	Linagliptin alone Mean	SD	Total	Weight	Mean Difference IV, Random, 95% CI
Emp 25 mg + Lin 5 mg vs Lin 5 mg	-2	4.17	134	-0.8	4.15	66	49.6%	-1.20 [-2.43, 0.03]
Emp 10 mg + Lin 5 mg vs Lin 5 mg	-2.7	4.14	135	-0.8	4.15	67	50.4%	-1.90 [-3.11, -0.69]
Total (95% CI)			269			133	100.0%	-1.55 [-2.42, -0.69]

$I^2 = 0\%$

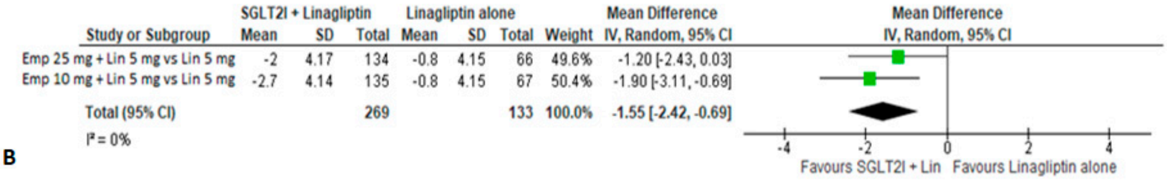

B

Figure S5. The mean change from baseline in: (A) HbA1c (%) (B) body weight (kg) between empagliflozin and linagliptin combination therapy versus linagliptin monotherapy in treatment naïve type 2 diabetes patients. SGLT2I, SGLT2 inhibitor; Emp, empagliflozin; Lin, Linagliptin.
